# The distance and chance of lifetime geographical movement of physicians in Japan: an analysis using the age-period-cohort model

**DOI:** 10.1186/s12960-018-0289-5

**Published:** 2018-06-13

**Authors:** Hiroo Ide, Shunsuke Doi, Hidenao Atarashi, Shinsuke Fujita, Soichi Koike

**Affiliations:** 1 Chiba University Hospital, Inohana 1-8-1, Chuo-ku, Chiba-shi, Chiba 260-8677 Japan; 2 The University of Tokyo Hospital, Hongo 7-3-1, Bunkyo-ku, Tokyo 113-8655 Japan; 3 Center for Preventive Medical Services, Chiba University, Yayoicho 1-33, Inage-ku, Chiba-shi, Chiba 263-8522 Japan; 4 Jichi Medical University, Yakushiji 3311-1, Shimotsuke-shi, Tochigi 329-0498 Japan

**Keywords:** Geography, Physician, Movement, Geographic information system (GIS), Age-period-cohort model

## Abstract

**Background:**

The uneven geographical distribution of physicians in Japan is a result of those physicians electing to work in certain locations. In order to understand this phenomenon, it is necessary to analyze the geographic movement of physicians across the Japanese landscape.

**Methods:**

We obtained individual data on physicians from 1978 to 2012 detailing their attributes, work institutions, and locations. The data are from Japanese governmental sources (the Survey of Physicians, Dentists, and Pharmacists). The total sample size was 122 150 physicians, with 77.5% being male and 22.5% female. After obtaining the data, we calculated the geographical distance of each physician’s movement by using geographic information systems software (GIS; ArcGIS, ESRI, Inc., CA, USA). Geographical distance was then converted into time distance. We compared the resulting median values through nonparametric testing and then conducted a multivariate analysis. Our next step involved the use of an age-period-cohort (APC) model to measure the degree of impact three points of data, experience (experience years), the historical and environmental context of the data (survey year), and physician cohort (registration year) had on the movement of each physician.

**Results:**

The ratio of female physicians who selected an urban area as their first working location was higher than that of male physicians. However, the selection of an urban area was becoming more popular as a first working location for both males and females as the year of data increased. The overall distance of geographical movement for female physicians was less than it was for male physicians. Physicians moved the greatest distance between their second and fourth years following license acquisition, at which point the time distance became shorter. The median time distance was 46 min in 2000 and 22 min in 2008. The physicians in our study did not move far from their first working location, and the overall distance of movement lessened in the more recent years of study. The median distance of movement after 20 years was 25.9 km for male physicians, and 19.1 km for female physicians. The results of the APC model indicated that the effects of experience years (age) gradually declined, that the survey year (period) effects increased, and that the registration year (cohort) effects increased initially before leveling off.

**Conclusions:**

The trends following the introduction of the new mandatory training system in 2004 may imply that the concentration of physicians in Japan’s urban areas is expected to increase. After 2000, the effect of that period on physicians explains their geographical movements more so than the factor of their age.

## Background

The topic of geographical distribution has attracted worldwide attention in the field of health workforce research. Factors affecting health workers’ decisions when choosing workplaces include personal origins and values, family and community aspects, working and living conditions, career-related aspects, financial aspects, and bounding or mandatory service [1–4]. These factors are common motivators for physicians considering both international and regional migration.

The geographic distribution of the health workforce has been a major issue in health policy in Japan; in particular, the geographic distribution of physicians has been a long-standing problem. A shortage in the number of physicians makes human resource policy more difficult; the number physicians per 1000 people in Japan was 2.36 in 2014, which is lower than in other OECD countries [5]. Several health workforce policies were introduced after the mid-2000s. The government increased the number of enrollees in medical schools from 7625 in 2007 to 9419 in 2018 (an increase of 24%). In addition, the government allowed medical schools to establish special enrolment spots for those willing to work at designated sites, including rural and remote areas, aiming to improve the equitable geographic distribution of physicians. The number of spots was 1010 in 2018, more than 11% of total enrollees. The policy was supported by moderate evidence [4, 6–8]. Then, in 2015, the government established a special committee on the demand and supply of the health workforce, including physicians. This committee recently released an interim report [9]. However, its recommendations were based on stakeholders’ opinions rather than scientific findings.

We address two problems in health workforce policy in Japan: the lack of understanding of the labor market and the lack of empirical studies. First, policy should be designed from the perspectives of both the supply side and the demand side. Compared to the supply side, however, demand-side analysis of health workforce has been insufficient [10]. Second, Japan needs more empirical studies. For example, many discrete-choice experiments have been conducted in developing countries to evaluate the effectiveness of policy intervention [11, 12]. A study in Vietnam revealed that providing equipment in facilities and long-term education are effective measures for recruiting physicians in rural areas [13]. To our knowledge, similar research has not been conducted in Japan.

To more constructively discuss health workforce policy, evidence is needed regarding how physicians move. The uneven geographical distribution of physicians is a result of the selection process that physicians undergo when choosing a working location. This selection process accompanies geographical movement. In order to understand the phenomenon of uneven geographical physician distribution, it is necessary to analyze the geographic movement of those physicians.

Several previous studies have examined the existence of geographical movement in a cross-sectional manner. For example, it was recorded that an average of 6.5% of obstetricians and gynecologists in the United States of America moved each year of their employment, and about 60% of obstetricians and gynecologists from the same study set moved over a 10-year period [14]. Young black male and international medical school graduates also tended to relocate during 2005 to 2015 [14]. In Japan, primary care physicians who practiced in a rural area in 1980 tended to continue practicing in any rural area in 2002. However, female and younger primary care physicians who practiced in a rural area in 1980 were more likely to have quit practicing in a rural area by 2002 when compared to male and elder primary care physicians [15]. Data on female and younger physicians were positive predictors of migration in the United States [16]. Studies in both Japan and the United States indicated that factors of gender and age can explain the geographical movements of primary care physicians.

The above studies revealed the relationship between geographical movement and the characteristics of a physician, such as gender, age, and ethnicity [14, 15]. In addition to those findings, further information was obtained about the relationship between historical and environmental contexts, the generation a physician belongs to, and the degree of geographical movement throughout a physician’s working life. This information will aid the development of effective heath workforce policy.

A physician’s gender has been discussed as an essential factor when examining their geographical movement. It has been reported that different preferences exist between female and male physicians with regard to working location and specialty selection [17–19]. In Japan, there is some concern that an increasing number of female physicians will result in more unevenness in specialty choice and geographical distribution. However, there is currently no available quantitative data for use in an examination of that concern.

The purpose of this study was to examine the degree of distance and the timing of the geographical movements of physicians practicing in Japan during the course of their careers. To express the degree of a physician’s movement, we calculated distance and time distance. Moreover, we distinguished the effect on a physician’s geographical movement by factors of gender, years of experience, the time of movement, and generational cohort. Based on the results of this study, we will discuss the current health workforce policy in Japan, and examine possible policies that will be effective in creating an even geographical distribution of physicians.

## Methods

Before gathering data, we obtained approval from the Japanese Ministry of Health, Labour and Welfare. We used individual data from the Survey of Physicians, Dentists, and Pharmacists to conduct the study. The data were gathered from surveys administered by the Japanese government every 2 years from 1978 to 2012, and all physicians surveyed were required to return responses. The response rate was considered to be around 90%. (The survey is mandatory. However, the government does not penalize physicians who do not respond.) [20] For the purposes of our study, we selected data from subjects who obtained a physician’s license on even years that occurred during the survey period. From those individual records, we used information describing the subject’s unique ID, sex, survey year, year of acquisition of physician’s license (registration year), and municipality of employment. Using unique ID as a key, we chronologically linked the data of each physician. We treated the year in which a physician’s license was obtained as the year of cohort for that physician. The survey year had an effect on all physicians who registered at a certain period; that is, the effect of time was noted. We calculated years of experience by subtracting a physician’s registration year from their survey year (experience years = survey year − registration year).

We took the mergers of local municipalities into account and accordingly adjusted the representative points of the municipalities as of 2010. The representative point was defined as the location of the municipal office during 2010. We subsequently used a geographic information system (GIS; ArcGIS, ESRI, Inc., CA, USA) to calculate the geographical distance between the municipality in which a physician’s initial workplace was located, and the locations where in which they subsequently worked. Geographical movement was expressed in terms of linear distance, road distance, and road distance converted into time distance. When expressed simply as linear distance or road distance, the costs for movement become abstracted. Therefore, road distance was converted into time distance and then standardized. Using the GIS, we converted distance into time distance by using the travel speeds stipulated by Japanese law in a given area. When converted to time, the upper limit was 240 min. Although younger physicians are more likely to relocate [15, 16], only around 5% of them relocated more than a 240-min distance from their previous location. The number of physicians that relocated within their first 2 years of employment was 6041. For physicians that relocated between 2 and 4 years of employment, the number was 7289.

We first classified the municipalities in which each doctor worked and then aggregated physician data by sex and registration year. For our purposes, the term “urban areas” refers to six of Japan’s prefectures (Tokyo, Kanagawa, Aichi, Kyoto, Osaka, and Fukuoka). When examining the uneven geographical distribution of physicians, the Japanese government has often defined the term in the same way. We categorized subjects by sex and displayed their median aggregated values as the sum of values over the life of a subject until the year 2012.

We selected physicians who registered from 1978 to 2008 and displayed the median values of their time distance between work locations every 2 years for the categories of registration year and sex. This is because the time distance that we calculated does not follow normal distribution. For physicians who registered from 1978 to 2002, we displayed the median, 25th percentile, and 75th percentile values of their time distance for the work locations of those physicians between their first and tenth years, categorized by sex. In the same manner, we displayed the values for years 0–20 for physicians who registered from 1978 to 1992. We then performed a multivariate analysis using a generalized linear model. There were some physicians who did not move during the initial 2-year period. Therefore, geographical movement was not distributed normally. We used log-transformed time distance as a dependent variable and assumed that the link function was of normal distribution. We selected sex and registration year as explanatory variables and then created dummy variables. After our analysis, we converted the coefficients into values of time distance.

In the above analysis, we employed the registration years as explanatory variables, and estimated the time distance for years 10 and 20 of the survey. In addition, it was probable that a physician’s experience years affected their geographical movement. A problem was identified during the simultaneous input of these three variables into statistical models. That is, an identifiability problem occurred, and the models omitted one of the three variables during process calculation. The following formula describes the relationship of those three variables in that context:

$$ \mathrm{experience}\ \mathrm{year}\mathrm{s}=\mathrm{survey}\ \mathrm{year}\mathrm{s}-\mathrm{registration}\ \mathrm{year} $$

Many outcomes could be affected by those three variables. For instance, the prevalence rate of cancer differs among generations. Age is an essential factor for the incidence of cancer. For instance, a generation of people may be exposed to harmful environmental factors such as chemical agents. As well, the infection rate for the hepatitis C virus is strongly related to a person’s birth cohort. The factors of age, period, and cohort are related to one another in similar fashion to the above formula. Therefore, a statistical model (the age-period-cohort model (APC model)) that can identify the effects of those three variables was developed, and the necessary computer power for the calculation of that model is readily available. Researchers can easily operate several types of the APC model by using statistical software.

The APC model assumes the existence of a certain effect (drift), measures the difference between drift and period as well as drift and cohort, and identifies the effects of the inputted variables. We used a method that incorporated a spline regression model, equipped on Stata 14.1 (StataCorp LP., TX, USA) [21, 22].

$$ {\displaystyle \begin{array}{c}N\sim \mathrm{Poisson}\left(\mu \right)\\ {}g\left(\frac{\mu }{\mathrm{exposure}}\right)={f}_a\left(\mathrm{age}\right)+{f}_p\left(\mathrm{period}\right)+{f}_c\left(\mathrm{cohort}\right)+\beta \mathrm{drift},\end{array}} $$

where *μ* means frequency of movement following Poisson distribution, *g* is a link function, *f*_*a*_, *f*_*b*_, *and f*_*c*_ are natural cubic splines, and *β* is the coefficient of drift. We used the APC model to examine the effects of experience years as age, survey years as period, and registration years as cohort against the ratios of physicians that changed work locations, and adjusted the numbers by sex.

A Student *t* test was used for the comparison of mean values. A chi-square test was used for the comparison of the ratio of the two groups, and a Mann-Whitney *U* test was used to compare median values. STATA was used for statistical analysis with a significance level of 5%.

## Results

**Fig. 1:**
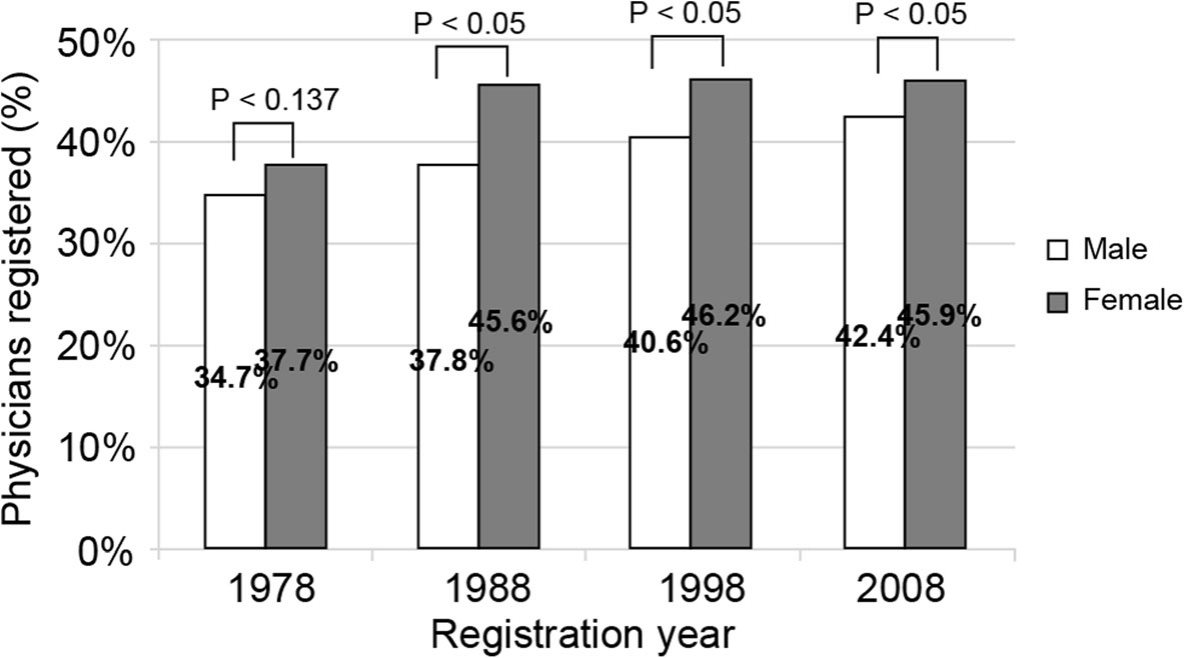
The percentages of physicians registered in 1978, 1988, 1998, and 2008 that selected a working location in an urban area during the first year of registration by sex. We conducted a chi-square test to determine the percentages between male and female physicians for each registration year

The number of target physicians who registered between 1978 and 2012 was 122 150. The number of female physicians was 27 423 (22.5% of the total). Physicians who registered in 1978, 1988, 1998, and 2008 constituted 5456, 7866, 7807, and 7728 of the group, respectively (Table 1). The ratio of female physicians who selected an urban area as their first work location was higher than that of male physicians. However, there was a chronological percentage increase for both male and female physicians who chose an urban area for a first work location. The ratio of female physicians who selected urban areas in 1978 was 37.7 and 34.7% (*p* < 0.05) for male physicians. Of those registered in 2008, the percentage of female physicians who selected urban areas was 45.9 and 42.4% (*p* < 0.05) for male physicians (Fig. 1).

**Table 1:** Number of study subjects by registration year and sex

Registration year	Total	Male	Female	Female percentage (%)
1978	5 456	4 833	623	11.4
1980	7 142	6 293	849	11.9
1982	7 566	6 594	972	12.8
1984	8 479	7 363	1 116	13.2
1986	7 958	6 767	1 191	15.0
1988	7 866	6 556	1 310	16.7
1990	7 873	6 414	1 459	18.5
1992	8 001	6 355	1 646	20.6
1994	7 988	6 062	1 926	24.1
1996	8 105	6 063	2 042	25.2
1998	7 807	5 813	1 994	25.5
2000	7 078	4 915	2 163	30.6
2002	7 900	5 473	2 427	30.7
2004	7 456	4 940	2 516	33.7
2006	7 747	5 216	2 531	32.7
2008	7 728	5 070	2 658	34.4
Total	122 150	94 727	27 423	22.5

**Fig. 2:**
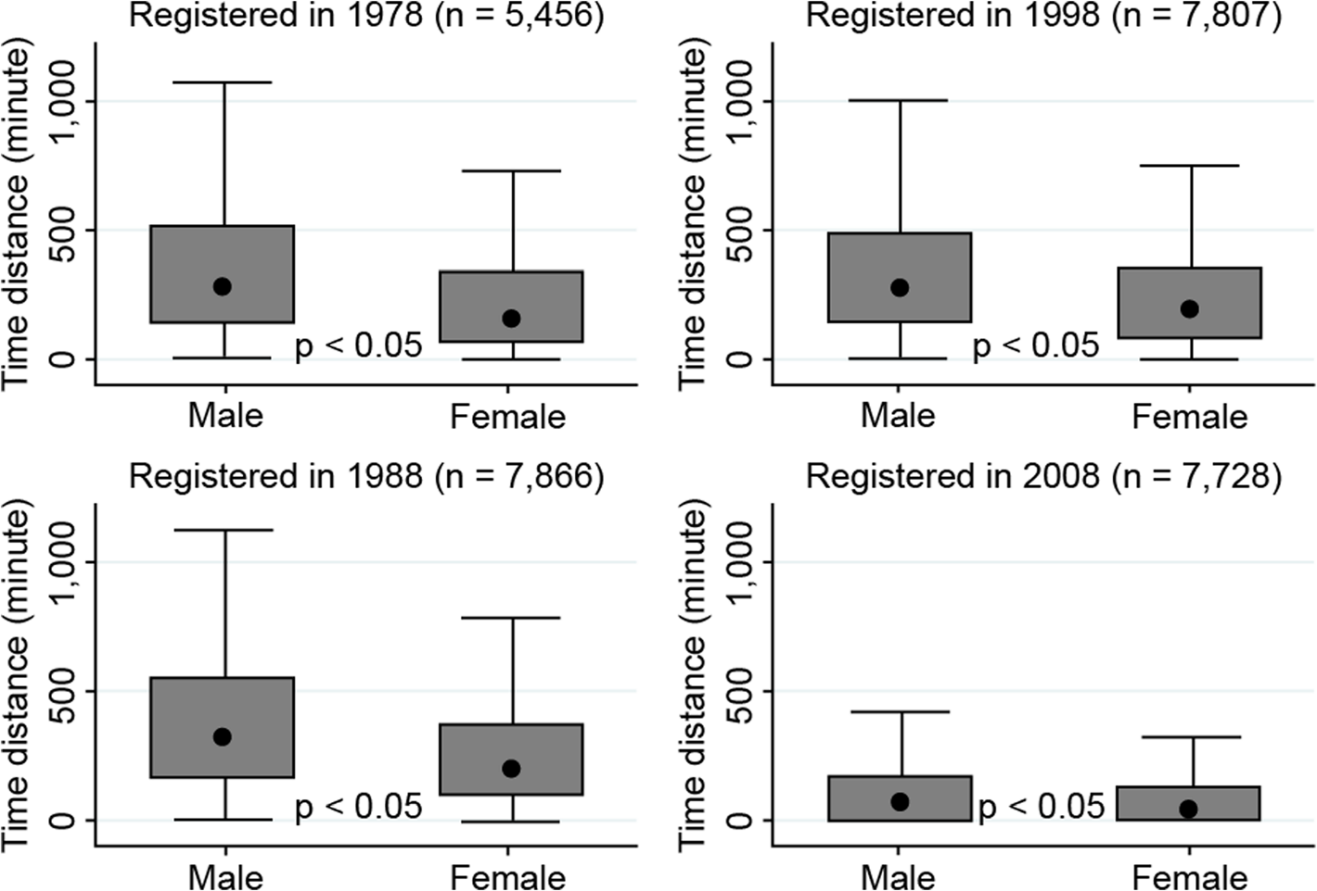
Accumulated value of time distance according to geographical movement until 2012 for physicians who registered in 1978, 1988, 1998, and 2008 by sex

Compared to male physicians, female physicians underwent less geographical movement (Fig. 2). For example, of the persons who obtained a physician’s license in 1978, the median time distance at the year 2012 was 265 min. The time distance for male physicians was 279 min and 158 min for female physicians (*p* < 0.05). These results were consistent in the four target cohorts of our analysis.

**Fig. 3:**
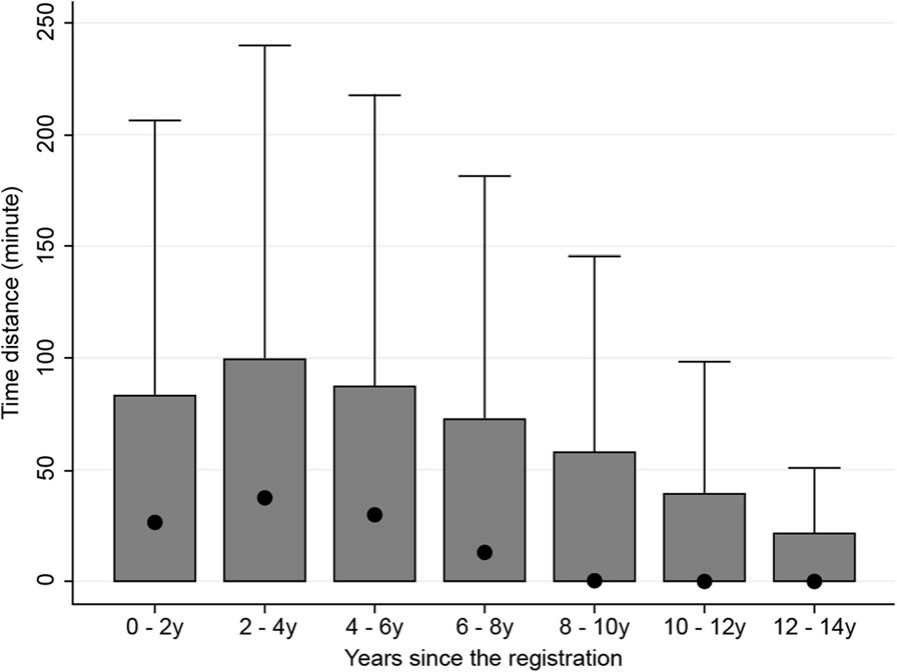
Time distance calculated according to the geographical movement of physicians every 2 years since registration. This figure shows time distance at 2-year intervals after registration up to 14 years (from the left to right). The dots represent median values; the upper lines of the boxes represent the 75th percentile, and the bottom lines represent the 25th percentile. We calculated these values from all samples, which numbered 122 150. The median values of time distance for every 2 years since registration up to 14 years registered at 0, except for 0 to 2 years (27 min), 2 to 4 years (37 min), 4 to 6 years (30 min), and 6 to 8 years (13 min). The median values for those four periods were significantly different (*p* < 0.05)

**Fig. 4:**
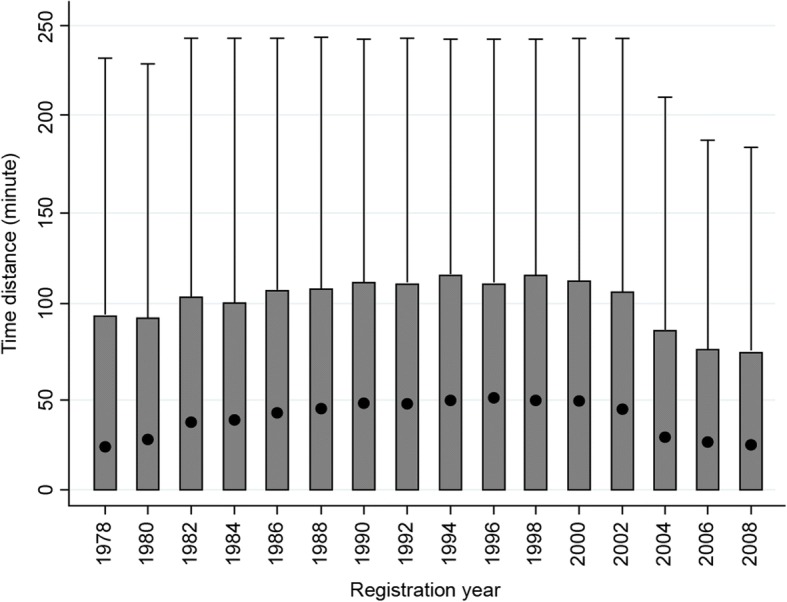
Time distance calculated according to the geographical movement of physicians from 2 to 4 years after the acquisition of a license by the cohorts of each registration year. The dots represent median values; the upper lines of the boxes represent the 75th percentile; and the bottom lines represent the 25th percentile

In this study, we measured geographical movement as shown by time distance of physicians every 2 years after the acquisition of a physician’s license. The period in which the largest quantity of geographical movement occurred was the 2-year period between years 2 and 4 (Fig. 3). Of the geographical movement that occurred during this period, the time distance of the movement was largest for those who registered as a physician around 2000. For years 2 to 4, the median time distance for physicians who registered in 2000 was 46 min. Although an increasing trend was observed until the year 2000, the time distance has become shorter in recent years. In the final cohort in which geographical movement from the second to fourth years could be measured (those who registered as physicians in 2008), the median number was 22 min (Fig. 4).

The median distance between the initial workplace and the workplace at 10 years after registering as a physician was 15.0 km in total. The distance was 15.6 km for male physicians, and 12.5 km for female physicians (*p* < 0.05). Similarly, the distance at the 20-year mark was 24.9 km in total. The distance was 25.9 km for male physicians and 19.1 km for female physicians (*p* < 0.05). These numbers suggest that physicians do not move great distances from their initial places of work over the duration of their career. The results obtained through the generalized linier model revealed that the distance of geographical movement of physicians at 10 years after acquiring a physician’s license was 0.70 km longer for physicians who registered in 2002 than it was for those who registered in 1978 (Table 2). Similarly, the distance at 20 years was 0.73 km longer for physicians who registered in 1992 than it was for those who registered in 1978 (Table 3).

**Table 2:** Results of the generalized linear model indicating the distance from initial workplace to place of work 10 years after license acquisition

		Estimated value (km)	95% confidence interval (km)	*p* value
Female (reference: male)		0.69	0.65–0.74	0.1690
Registration year (reference: 1978)	1980	1.07	0.91–1.27	0.4020
	1982	0.94	0.79–1.11	0.4400
	1984	0.72	0.61–0.85	0.0000
	1986	0.87	0.74–1.02	0.0810
	1988	0.72	0.61–0.85	0.0000
	1990	0.81	0.69–0.96	0.0130
	1992	0.87	0.74–1.03	0.1030
	1994	1.06	0.90–1.25	0.4680
	1996	0.97	0.82–1.14	0.7020
	1998	0.87	0.74–1.03	0.1040
	2000	0.84	0.71–0.99	0.0350
	2002	0.70	0.59–0.83	0.0000

We calculated the distance between the initial and the 10-year workplace since registration before the statistical analysis. The last registration year included in the current analysis was 2002 because the most recent data was from 2012. The estimated values in the table were converted from the coefficients analyzed by the generalized linear model

**Table 3:** Results of the generalized linear model for the distance from initial workplace to the place of work 20 years after license acquisition

		Estimated value (km)	95% confidence interval (km)	*p* value
Female (reference: male)		0.68	0.61–0.75	0.0000
Registration year (reference: 1978)	1980	1.19	1.02–1.40	0.0320
	1982	1.06	0.91–1.25	0.4370
	1984	0.87	0.75–1.02	0.0860
	1986	0.98	0.84–1.15	0.8340
	1988	0.77	0.66–0.90	0.0010
	1990	0.74	0.63–0.86	0.0000
	1992	0.73	0.62–0.85	0.0000

We calculated the distance between the initial workplace and place of work 20 years after registration, before the statistical analysis. The last registration year included in the current analysis was 1992 because the most recent data was from 2012. The estimated values in the table were converted from the coefficients analyzed by the generalized linear model

**Fig. 5:**
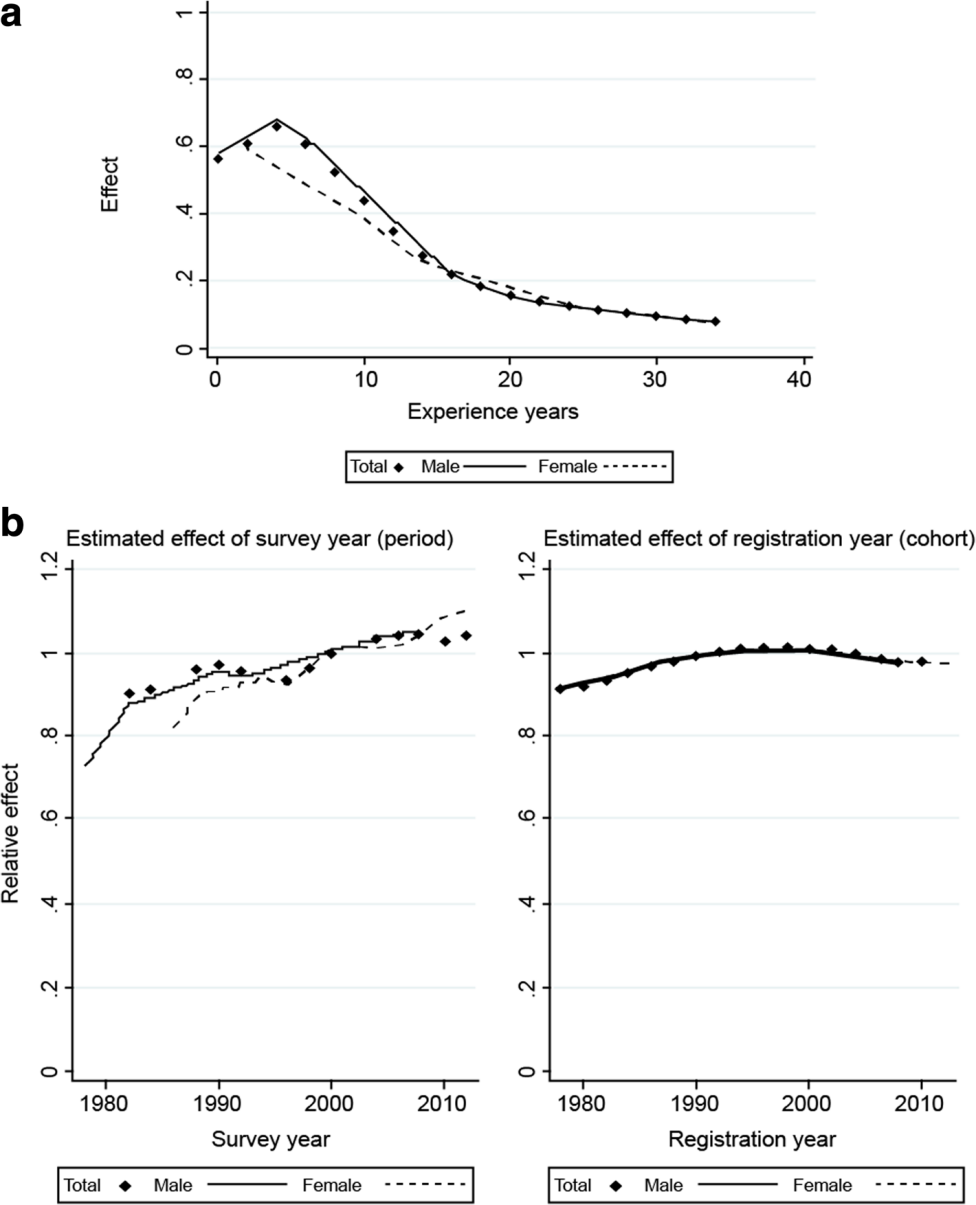
The relative effects of experience years, survey year, and registration year on the chances of the geographic movement of a physician’s workplace for males and females. **a** Effect of experience years (age). **b** Relative effects of survey year (period) and registration year (cohort). These figures are depicted based on the results of the age-period-cohort model. In that model, experience years, survey year, and registration year were treated as age, period, and cohort, respectively

The APC model’s results indicate that the effect of experience years (age) was initially very pronounced, but decreased as experience years accumulated, as illustrated in Fig. 5. Female physicians with less experience years were less likely to move when compared to male physicians. Figure 5 also indicates that the effects on the chances of movement for survey year (period) and registration year (cohort) influence each other. The chronological effect of the survey year increased positively, but the effect of registration year increased very little before nearly leveling off after the year 2000.

## Discussion

This study quantitatively examined changes in the workplace locations of physicians, as well as the relationship of that change to a physician’s attributes and timing. The government of Japan has considered the geographic distribution of physicians based on the number of physicians in prefectural and municipal areas. This is an easy method, but it is too imprecise to describe the facts of distribution. For example, as an example of uneven geographic distribution, the government pointed out that the number of physicians per capita in the largest prefectures was twice that of the smallest prefectures [9]. GIS is more widely used now than ever before, and this technology can help us to quantitatively understand the geographic distribution of physicians.

This study examined the movement of physicians over time. Here, we propose a novel approach for analyzing the unevenness of geographical physician distribution. The results of this study revealed that, on average, physicians moved between 10 and 30 km from their first location of employment at the 10 and 20 year marks since becoming a physician. This does not seem to be a great distance of movement. Physicians tend to maintain a connection with the locations in which they were first employed. Additionally, the geographical movement of female physicians was found to be smaller than that of male physicians. It may be a more difficult task to convince female physicians to relocate from urban areas than to convince male physicians for the purpose of ensuring an adequate labor force.

There is a recent tendency for male physicians to concentrate in urban areas. Using our study as a reference, the ratio of aspiring female physicians has remained stagnant (31.5% in 2016). Moreover, when the employment rate of physicians is taken into account by examining physician age, the ratio of female physicians in 2035 is expected to remain at 30% [23]. Although it is true that female physicians have tended to concentrate in urban areas, the effects of female physicians on the overall geographical distribution of physicians in the future will likely be of less impact. Trends in the employment of male physicians have driven the phenomenon of urban concentration. The urban population was 34.4% in 1996, but had increased to 36.6% by 2014 (an increase of 2.4%) [24]. For physicians, the percentage increased from 39.1 to 43.8% during the same period (an increase of 4.7%). The concentration of physicians exceeded the percentage of total population in urban areas both in recent levels and in the change that occurred from 1996 to 2014. Government agencies predict that the concentration of Japan’s population into urban areas is expected to increase from 2010 to 2040 [25]. Physicians may expect that the demand for medical services is going to increase in urban areas, which is reflected by their geographical movements. It is our opinion that the general choice of work location for physicians is a rational one.

Furthermore, medical school entrance examinations have become more difficult, which may present a relative advantage for persons originating in urban areas. Many studies have reported that in order to increase the number of physicians in rural and remote areas, it is effective to educate persons from those regions as physicians and to subsequently enable them to develop work experience as early as possible [26–28]. Matsumoto et al. noted that a policy executed at a medical school in Japan, which was similar to the national policy launched in 2004, was effective for retaining physicians in their home prefecture [29]. Adding to previous knowledge, our results suggest that Japan’s recent selective enrolment policy, where students are obliged to work in a designated place for several years and receive a loan deduction, will be effective. As we have shown, once physicians choose an underserved area, they are not likely to move very far from their first workplace throughout their professional career. In addition, to mitigate geographic imbalance, our results suggest it might be effective to plan the first workplaces for all newly registered physicians. This policy would not regulate *freedom of movement*, but we can assume physicians will not move very far from their first workplaces. One advantage is that the subjects of this policy include 9000 newly registered physicians per year. The government could thus achieve geographic equity more rapidly than with the present selective enrolment policy, whose target is only 1000 physicians. Although a controversial point could concern how we plan the first workplace in view of attributes, motivation, current situations, future prospects, and so on, some developed countries have already introduced similar institutions.

Simultaneous to the development of a quota system for medical schools in 2004, the clinical training system became mandatory [30, 31]. Under the new clinical training system, a 2-year training period was required for physicians. The mandatory clinical training system seems to have affected the patterns of geographical physician movement. Under the new system, the number of trainee spots offered by hospitals has increased. As a result, the number of physicians who are able to select hospitals in urban areas has risen.

It is possible that the mandatory clinical training system has a counter-effect on the governmental policy of assigning enrolment spaces to physicians from rural areas. This is interesting to note, since both measures were implemented at the same time. The results of this study revealed that the distance of movement was greater for individuals who became physicians before 2002. Because male physicians tend to select urban areas as locations for starting their careers, and do not move far from those locations, the overall tendency of physicians to concentrate in urban areas is expected to increase. However, if excessive competition occurs for physicians in urban areas, they can theoretically relocate [32].

In this study, the survey year (period) is beginning to have a significant effect on the chances of a physician’s geographical movement. The survey year is affected by historical and environmental contexts, and also affects each physician equally. Given this result, future human resource policies aimed at the geographic induction of physicians may not need to differentiate according to the registration year (cohort) of physicians. Regarding the effects of age on the movement of physicians, there is a greater tendency for younger physicians to relocate. Why is the effect of period on the geographical movement of physicians increasing? Based on the data that we used, it is impossible to directly ascertain the reasons for the geographic movement of physicians. However, we can present some possible explanations. For example, the aging of physicians explains their movement, as shown by the APC model, and the aging of the population has effects from the demand side. Japan’s population has been gradually aging, with 27% of the population over age 65 in 2017. Population aging seems to appear as the effect of period in the APC model by increasing the demand for healthcare services. Furthermore, developments in transportation networks and similar lifestyle preferences, such as work and living locations, may have influenced the geographical movement of physicians. These potential factors are all related to social environment. In other words, those factors are expressed as the effect of period in the APC model.

Developments in infrastructure likely affect access to healthcare services. Physicians are more inclined to choose urban areas than the general population [4]. Our thinking about improving the geographic distribution of the health workforce has focused on how to settle physicians in underserved areas. Ultimately, improving access to health services is essential. Japan has constructed road transportation networks all over the country, and while the total length of general public roads has been mostly static, the length of express roads has increased by about 50% in the past 20 years. High-speed transportation systems can improve access to healthcare services by moving people more quickly. Physicians can commute from urban areas to rural areas more easily, and patients can have the opportunity to see more available physicians.

This study has several limitations. First of all, its intention was to examine the reasons physicians work in certain locations, and to explore the reasons they relocate. However, the reasons for their geographical movement remain unclear because the data is retrospective and does not contain information on the potential factors that are affecting their tendency to choose a work location, such as income, or lifestyle [1, 2]. This study uses time to explain the geographical movement of physicians. Because this factor appears to be important for ensuring the necessary number of physicians working in each region, the future elucidation of the reasons behind geographical movement is necessary. Additionally, in order to measure the geographical movement of physicians, a representative point of the municipality in which the physician worked was substituted, but this is an inaccurate way of exploring the data. In a previous study, we revealed that municipalities with especially small populations and large areas expressed apparent differences when the same method was used [33]. Therefore, there is a possibility that this study overestimated the geographic movement of physicians working in rural regions with large areas. When of all the available data is encoded by using GIS, it will be possible to calculate the exact distances of physician movement. However, because the quantity of data was large, it was not possible to use that method. Finally, physician age was substituted for experience in the APC model because age and experience years are highly correlated. However, the use of age in the model makes the purpose of the analysis easier to understand.

## Conclusions

In this study, we used government-sourced data from a multi-year survey involving all physicians registered in Japan between 1978 and 2012 to quantitatively examine the changes in the workplaces of those physicians and to explore the relationship of physician attributes and timing. The concentration of physicians in urban areas is especially notable for male physicians. Based on recent trends in geographical movement, the concentration of physicians in urban areas is expected to increase. Physicians generally maintain close geographical proximity to the locations in which they were first employed. During their early careers, physicians tend to move large distances. However, the distance of movement has become smaller in recent years, and the commonality among cohorts has increased. The authors of this study are of the opinion that a longitudinal analysis of geographical movement (not only in a cross-sectional manner) can contribute to the creation of governmental policies that have the potential to balance the uneven geographical distribution of physicians. This is because a longitudinal analysis provides more information about the trends in geographical movement regarding the projection of geographical physician distribution.

## Abbreviations

APC model: Age-period-cohort model
GIS: Geographic information system
